# Bacterial characterization of Beijing drinking water by flow cytometry and MiSeq sequencing of the 16S rRNA gene

**DOI:** 10.1002/ece3.1955

**Published:** 2016-01-18

**Authors:** Tingting Liu, Weiwen Kong, Nan Chen, Jing Zhu, Jingqi Wang, Xiaoqing He, Yi Jin

**Affiliations:** ^1^ College of Biological Sciences and TechnologyBeijing Forestry UniversityP. O. Box 162Beijing100083China

**Keywords:** Drinking water quality monitoring, flow cytometry, fluorescence fingerprints, MiSeq sequencing, opportunistic pathogenic bacteria

## Abstract

Flow cytometry (FCM) and 16S rRNA gene sequencing data are commonly used to monitor and characterize microbial differences in drinking water distribution systems. In this study, to assess microbial differences in drinking water distribution systems, 12 water samples from different sources water (groundwater, GW; surface water, SW) were analyzed by FCM, heterotrophic plate count (HPC), and 16S rRNA gene sequencing. FCM intact cell concentrations varied from 2.2 × 10^3^ cells/mL to 1.6 × 10^4^ cells/mL in the network. Characteristics of each water sample were also observed by FCM fluorescence fingerprint analysis. 16S rRNA gene sequencing showed that Proteobacteria (76.9–42.3%) or Cyanobacteria (42.0–3.1%) was most abundant among samples. Proteobacteria were abundant in samples containing chlorine, indicating resistance to disinfection. Interestingly, *Mycobacterium*,* Corynebacterium,* and *Pseudomonas*, were detected in drinking water distribution systems. There was no evidence that these microorganisms represented a health concern through water consumption by the general population. However, they provided a health risk for special crowd, such as the elderly or infants, patients with burns and immune‐compromised people exposed by drinking. The combined use of FCM to detect total bacteria concentrations and sequencing to determine the relative abundance of pathogenic bacteria resulted in the quantitative evaluation of drinking water distribution systems. Knowledge regarding the concentration of opportunistic pathogenic bacteria will be particularly useful for epidemiological studies.

## Introduction

Water treatment systems normally have multiple protection systems to prevent microbial contamination (WHO, 1999). Drinking water is delivered to the consumer through distribution systems, and these systems need to maintain water quality. Disinfectant components are contained within the distribution network to inhibit microbial growth; however, microbial quality may change due to changes in temperature, flow velocity, residence times, sediments, biofilms, and potentially, the intrusion of untreated water from the subsurface, disinfectant depletion, and nutrient contamination (Lautenschlager et al. 2010, 2013). Monitoring distribution systems for bacterial growth is therefore important to ensure public health.

Heterotrophic plate count (HPC), which is based on the detection of bacteria by culture media utilization, represents a standard tool used to assess general microbiological diversity (Sartory 2004; Uhl and Schaule 2004). However, drinking water is well‐known to carry only a small number of bacteria that readily grow on conventional nutrient media (Gillespie et al. 2014). In practice, some bacteria enter “uncultivable” or “viable‐but‐not‐cultivable” (VBNC) states when outside pressure, temperature, nutrition, and other conditions change, but these bacteria can become active again under suitable conditions. This indicates that bacterial contamination can be overlooked when using the HPC method, which may consequently increase risk.

Use of flow cytometry (FCM) to assess total and intact cell concentrations in drinking water has been described previously (Hoefel et al. 2003; Hill et al. 2005; Lautenschlager et al. 2010, 2013; Besmer et al. 2014; Prest et al. 2014), and was used in this study due to the ease of the protocol and rapid (results obtained in 15 min), highly reproducible (<5% error) and sensitive (detection change of ≤3% of initial value) results (Lautenschlager et al. 2013). Fluorescent stains, such as propidium iodide (PI) and SYBR Green I preferentially bind to nucleic acids, making it possible for FCM to measure bacterial concentrations. These straightforward parameters are useful for monitoring treatment processes and detecting changes in drinking water quality (Vital et al. 2010; Prest et al. 2013). The determination of total and intact cell counts using FCM was recently standardized via an interlaboratory ring trial, and was officially accepted as a guideline method for drinking water analysis in Switzerland (SLMB, 2012). Moreover, based on distinctly different fluorescence intensities, bacteria have been broadly classified into two groups: low nucleic acid content (LNA) bacteria and high nucleic acid content (HNA) bacteria, thereby creating a bacterial community “fingerprint” (De Roy et al. 2012; Hammes et al. 2012) unique to each sample and dependent on the bacterial community composition and DNA content (De Roy et al. 2012; Koch et al. 2013). Therefore, fingerprint information can be valuable for the detection of composition changes that are not reflected in cell concentration measures.

The 454 pyrosequencing platform has been widely used in previous studies for 16S rRNA sequencing due to its read length advantages. The development of PE250 and PE300 sequencing strategies using the MiSeq platform has increased read length and improved species detection accuracy (Luo et al. 2012). Due to its highly accurate sequencing and ability to identify species that are in low‐abundance at low cost, this technique is now the preferred method for researching microbial diversity (Caporaso et al. 2012; Degnan and Ochman 2012). Using the Illumina MiSeq sequencing platform, samples in the present study were sequenced by the paired‐end method, and a small fragment library was constructed. Using this method, microbial community composition (identity) and structure (proportion) were revealed using mosaic‐filtered and operational taxonomic unit (OTU) clustering, and species abundance was noted and analyzed. In addition, the microbial composition was further analyzed by alpha and beta diversity. Sequencing can therefore provide comprehensive and qualitative information on drinking water distribution pipeline ecology.

Combining highly quantitative FCM data with detailed sequencing could provide a promising tool for both monitoring and detailed investigations of distribution systems. Several studies have applied both FCM and sequencing, such as monitoring the variations in bacterial community characteristics in full‐scale distribution network (Prest et al. 2014), investigating how water treatment affected both bacterial abundance and diversity (Hoefel et al. 2005) or performing lab‐scale batch experiments under controlled conditions (Bombach et al. 2011). These studies showed that the changes in number of special bacteria could be detected using the combined methods.

The objective of this study was to evaluate the combination of FCM bacterial cell counting, fingerprinting, and MiSeq sequencing data for the detection and characterization of microbes in drinking water distribution systems in Beijing. Unlike previous studies, in this study, additional analyses were performed comparing HPC with FCM detection methods, and combining MiSeq sequencing to produce novel information regarding water sample contamination at different geographical locations and distribution systems.

## Materials and Methods

### Sampling

**Figure 1:**
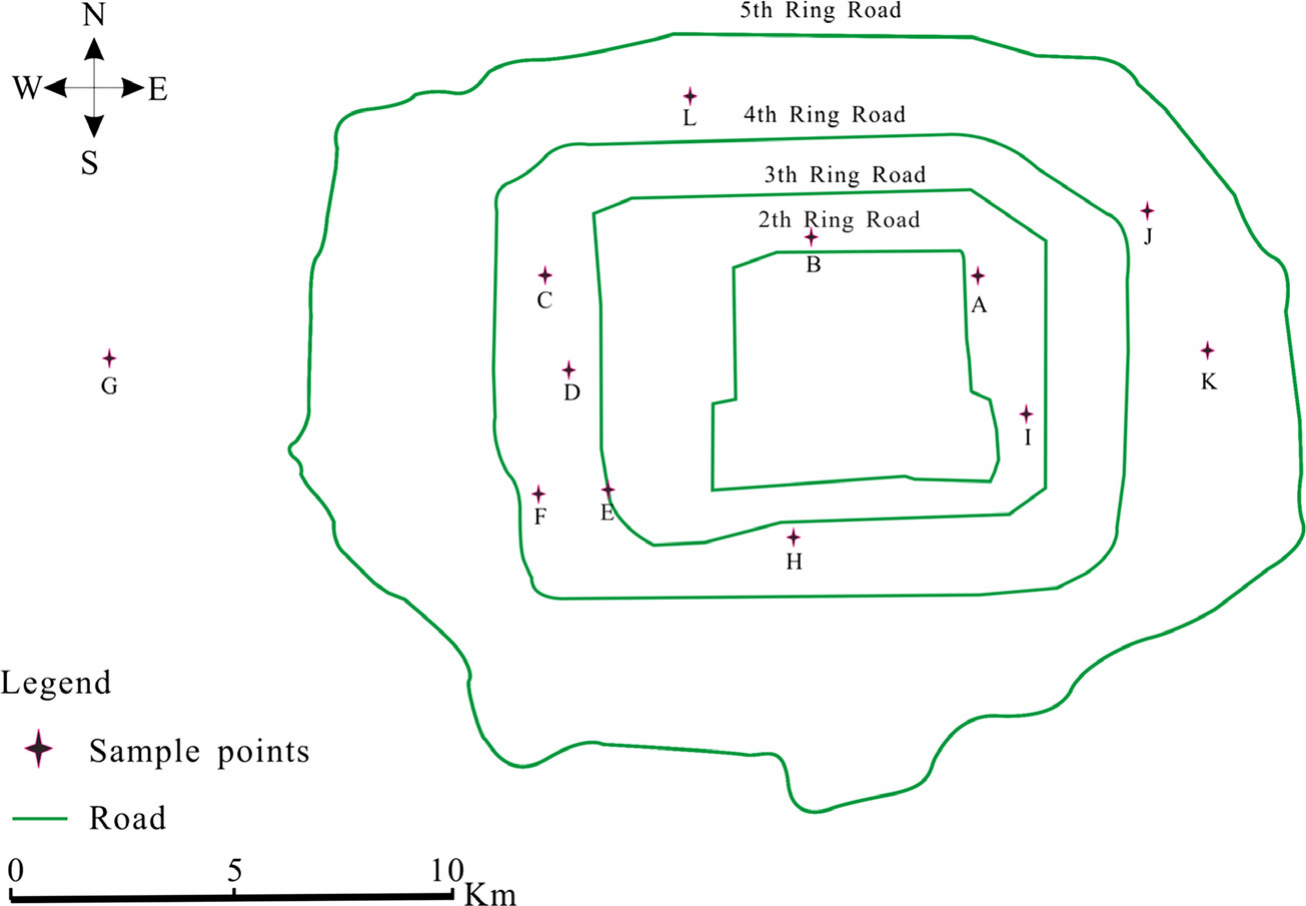
Sampling sites of drinking water in Beijing.

Twelve water samples were taken (assigned letters from A–L) from the Beijing water distribution network, as shown in Figure 1. The source water of sample L came from SW while those of others came from GW. The sites from which water samples were taken are usually monitored by the appropriate government agency. Based on the results detected by relevant departments, culturable coliforms, physical and chemical indicators of 12 water samples were in line with GB5749‐2006. For example, culturable coliforms were 0 in 100 mL, 0.05 ≤ residual chlorine ≤ 0.60 mg/L, 0.24 ≤ oxygen consumption (COD_Mn_) ≤ 1.80 mg/L and 0.08 ≤ turbidity ≤ 0.93 NTU (http://www.bjwatergroup.com.cn/). Sampling was performed according to the Scottish Water sampling procedures. Briefly, taps were flushed for 3 min and flame‐sterilized using a blow torch for 30 sec; after flushing the tap for an additional 30 sec, water was sampled into sterile 200 mL sample bottles containing a final sodium thiosulfate (Sigma‐Aldrich, Shanghai, China) concentration of 0.8 g/L (GB5749‐2006) to eliminate residual disinfectants. Samples were stored and transported in a refrigerated van at 4°C and analyzed within 24 h of sampling by FCM and HPC. All samples were performed in triplicate. An additional 100 L of water was sampled and used to analyze microbial community structure.

### Flow cytometry (FCM)

For the determination of total cell concentrations, water samples were stained according to the standardized protocol described in the Swiss guidelines for drinking water analysis. Briefly, SYBR Green I stock (Invitrogen, Basel, Switzerland) was diluted 100‐fold in anhydrous dimethylsulfoxide (DMSO; Sinopharm, Shanghai, China) to obtain a working solution. Samples (1 mL) were preheated to 35°C (5 min), stained with 10 *μ*L/mL SYBR Green I, and incubated in the dark for 10 min at 35°C prior to measurement. To assess intact cell concentrations, propidium iodide (PI; 30 mmol/L, Sigma‐Aldrich) was mixed with SYBR Green I working solution to a final PI concentration of 0.6 mmol/L. Ten *μ*L of the dye mix was then added to 1 mL of water. Following incubation, 100 *μ*L samples were analyzed using a BD Accuri C6 flow cytometer equipped with a 50 mW laser emitting at a fixed wavelength of 488 nm. Data analysis was performed using BD Accuri CFlow^®^ software (Becton, Dickinson and Company, New Jersey). Green fluorescence was collected in the FL1 channel at 533 nm, and red fluorescence in the FL3 channel at 670 nm, with the trigger set on green fluorescence. No compensation was required. Fingerprint analysis was based on separation of the two clusters formed by low (LNA) and high (HNA) nucleic acid content bacteria in FCM dot‐plots of FL1 (an indicator of apparent cellular nucleic acid content) and sideward scatter (SSC, an indication of cellular size), as shown in Figure S1. Quantification and fingerprint comparison from different water samples were acquired using the percentage of two clusters and relative nucleic acid content (calculated from the green fluorescence distribution). All measurements were performed in triplicate.

### Heterotrophic plate count (HPC)

The HPC method was performed using R2 agar (R2A, Hope Bio‐Technology, Qingdao, China), because R2A has been previously shown to result in higher colony counts than the conventional agar recommended by the Swiss guidelines for drinking water (Uhl and Schaule 2004). Samples were inoculated on nutrient‐poor R2A plates using the spread plate technique (Uhl and Schaule 2004). All measurements were performed in triplicate. All plates were incubated in the dark at 30°C for 7 days, after which, colony forming units (CFUs) were counted manually.

### Ultrafiltration (UF)

UF used for concentrating and recovering microbes from large volumes of water, has been described previously (Hill et al. 2007; Polaczyk et al. 2008; Liu et al. 2012a; Mull and Hill 2012). Compared to conventional methods, this technique is faster and more efficient at concentrating viruses, bacteria, and parasites. Due to their small pore sizes, UF membranes are capable of concentrating microbes based on size exclusion (Hill et al. 2007). Water samples (100 L) were collected in HD‐PE bottles containing sodium thiosulfate. Each sample was filtered by hollow‐fiber UF (Hemodialyzer, Rexeed‐25S, Asahi KASEI) with sodium polyphosphate (Sigma‐Aldrich) and surfactants Tween 80 (Sinopharm, Shanghai, China) within 4 h of sampling. UF procedures were performed as described previously (Hill et al. 2005, 2007; Polaczyk et al. 2008; Liu et al. 2012a). The filtration unit configuration is shown in Figure S2. One hundred liters of tap water was concentrated to 250 mL in 2 to 3 h, depending on the experimental conditions (Hill et al. 2005). The concentrated solution (250 mL) was then filtered through a 0.2 *μ*m pore size isopore membrane filter (Merck Millipore, Billerica, MA). Filter membranes were stored at −20°C until processing. Genomic DNA was extracted from the collected biomass using a Fast DNA SPIN Kit (MP Biomedical, Santa Ana, CA) according to the manufacturer's instructions.

### Bacterial community analysis with 16S rRNA gene sequencing

Bacterial 16S rRNA genes were amplified variable region 4 of the bacterial 16S rRNA gene using the following bacteria‐specific primers: forward, ‘515F,’ 5′‐GTGCCAGCMGCCGCGGTAA‐3′ and reverse, ‘806R,’ 5′‐GGACTACHVGGGTWTCTAAT‐3′ (Wagner et al. 2014). A single‐step 30 cycle polymerase chain reaction (PCR) using Phusion^®^ High‐Fidelity PCR Master Mix (New England Biolabs, Ipswich, MA) was performed for each DNA sample. PCR was performed using the following parameters: initial denaturation at 98°C for 1 min, followed by 30 cycles of denaturation at 98°C for 10 sec, annealing at 50°C for 30 sec, and elongation at 72°C for 60 sec, with a final extension step at 72°C for 5 min. PCR products were detected on 2% agarose gels. Samples with a bright main strip between 400 and 450 bp were chosen for further experimentation. Sequencing libraries were generated using an NEB Next^®^ Ultra^™^ DNA Library Prep Kit from Illumina (New England Biolabs, Ipswich, MA) following the manufacturer's recommendations. Library quality was assessed using a Qubit^®^ 2.0 Fluorometer (Thermo Scientific, Massachusetts) and Agilent Bioanalyzer 2100 system. Lastly, the library was sequenced using an Illumina MiSeq platform, and 250/300 bp paired‐end reads were generated.

### Sequencing data analyses

Paired‐end reads from the original DNA fragments were merged using FLASH (V1.2.7, http://ccb.jhu.edu/software/FLASH/; Magoč and Salzberg 2011), and assigned to each sample according to the unique barcodes. Sequence analyses were performed using the Uparse software package (Uparse v7.0.1001, http://drive5.com/uparse/; Edgar 2013) and Uparse‐OTU and Uparse‐OTU ref algorithms. In‐house Perl scripts were used to analyze alpha (within samples) and beta (among samples) diversity. Sequences with ≥97% similarity were assigned to the same OTU. Representative sequences for each OTU were chosen, and the RDP classifier (Version 2.2, http://sourceforge.net/projects/rdp-classifier/; Wang et al. 2007) was used to annotate taxonomic information for each representative sequence. In order to compute alpha diversity, the OTU table was rarified, and three metrics were calculated: Chao1, which estimates the species abundance; observed species, which estimates the number of unique OTUs found in each sample; and the Shannon index. Rarefaction curves were generated based on these three metrics. Graphical representation of the relative bacterial abundance from phylum to species was visualized using a Krona chart. Cluster analysis was preceded by principal component analysis (PCA), which was applied to reduce the dimension of the original variables using the Qiime software package (Illumina, California).

## Results

### Bacterial concentrations and flow cytometry fingerprints of 12 water samples taken from the water distribution network

**Figure 2:**
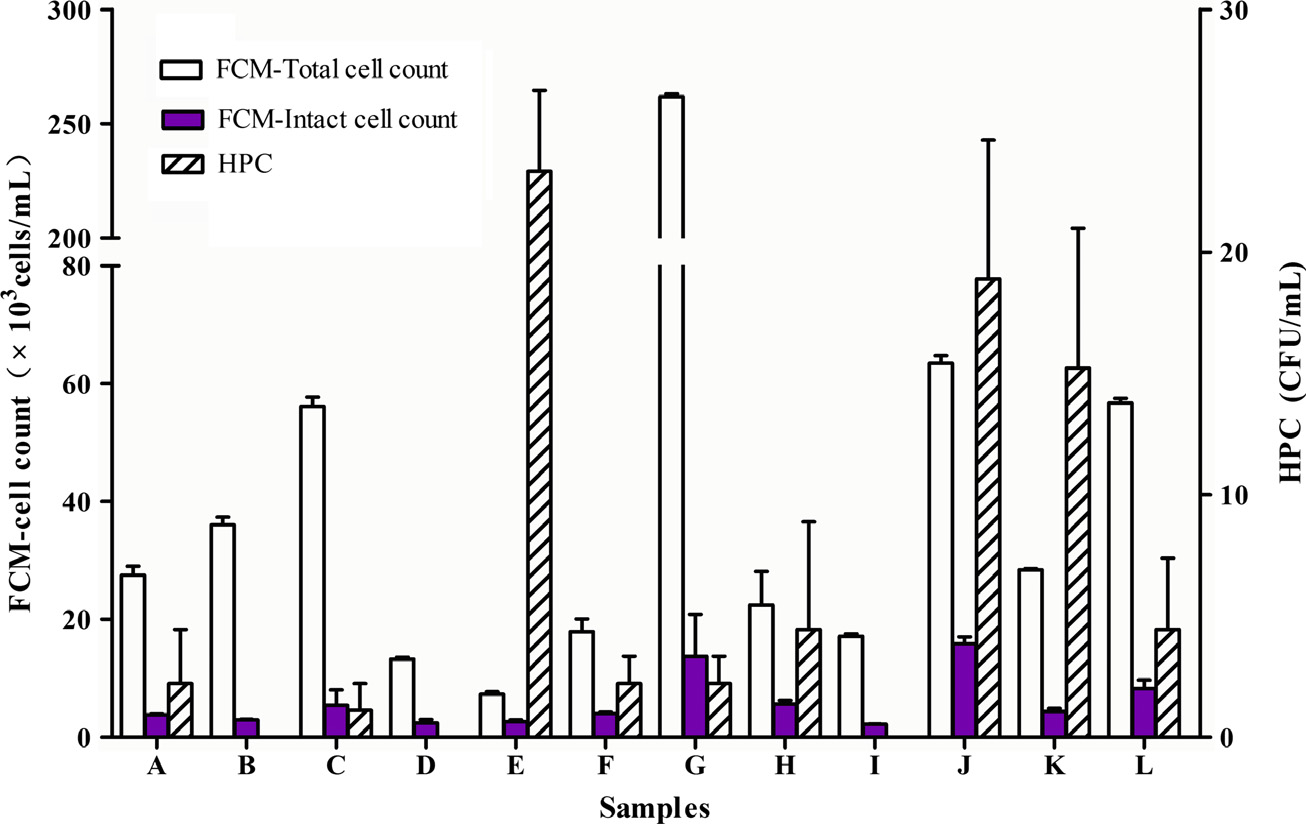
Bacterial concentrations of the 12 drinking water samples by two methods.

Flow cytometry total cell concentrations varied from 7.3 × 10^3^ cells/mL to 2.6 × 10^5^ cells/mL, and intact cell concentrations varied from 2.2 × 10^3^ cells/mL to 1.6 × 10^4^ cells/mL (Fig. 2) within the distribution network. Sample J contained the largest number of intact cells, followed by samples G and L, while sample I contained the lowest number. With respect to plate count, the highest values were detected in sample E (23 CFU/mL vs. 2 CFU/mL in samples A, F, and G, and 0 CFU/mL in samples B, D, and I). However, this method was unable to distinguish between the same results samples. According to GB5479‐2006 requirements, total CFU cannot exceed 100 per mL; 12 water samples were qualified because the HPC results varied from 0 to 23 CFU/mL. To a certain extent, HPC appeared to underestimate the number of microorganisms in drinking water and then we conclude that the tested water contained several bacteria that had previously gone undetected.

**Figure 3:**
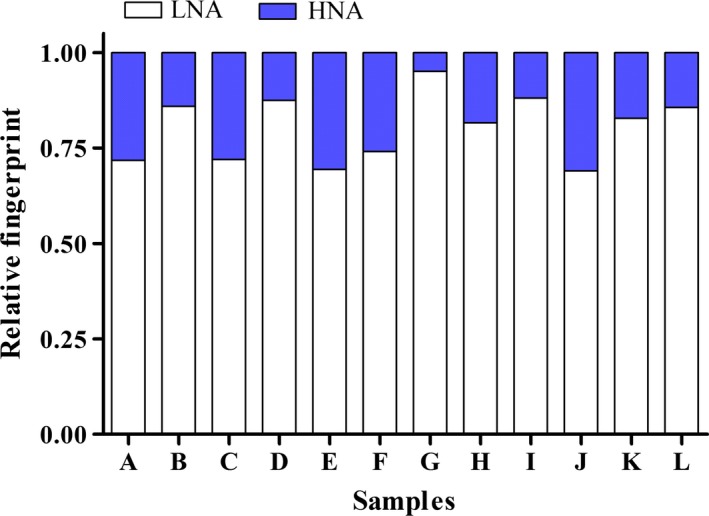
Comparison of bacterial flow cytometry fingerprints from 12 drinking water samples.

Microbial concentration differences determined by FCM were clearly related to FCM fingerprint differences in the 12 water samples tested. Figure 3 showed that the 12 samples displayed distinct FCM fingerprints. The separation between LNA and HNA clusters was at approximately the same fluorescent intensity (for our flow cytometer, around 6 × 10^4^ a.u.) in all samples, and was used as the experimental basis for selecting LNA and HNA gating positions (Figure S1). Figure 2 showed that the number of intact cells consisted of ≤50% of the total cell number. Figure 3 showed that HNA cells were <50%. This result was similar to what Wang et al. (2009) said most of HNA bacteria are considered active and LNA bacteria are inactive. FCM fingerprinting is a rather simplistic approach used to provide information regarding bacterial community characteristics that cannot be obtained by FCM cell counting alone.

### Characteristics of MiSeq sequencing results

Biodiversity of the 12 water samples was investigated by analyzing OTUs, Chao 1, and Shannon indices at cut‐off levels of 3% and 5% (Table 1). The Chao1 estimators were calculated to show species richness among the samples. Chao1 numbers were considerably higher than OTU numbers, suggesting that more OTUs may exist in bacterial communities. The Shannon index was also generated to assess community diversity. OTU, Chao1 and the Shannon index revealed that sample L originating from SW had the highest bacterial diversity of the 12 samples. This result was supported by the rarefaction curves of the 12 samples at the 3% distance cut‐off level, which revealed species richness variations in the drinking water distribution systems. In addition, rarefaction curves directly reflected sequencing results. Rarefaction curves of the 12 samples continued to climb and did not reach an asymptote at the 3% distance cutoff level (Figure S3), suggesting that greater bacterial diversity was present, and highlights the need to further investigate the communities. In contrast, the Shannon diversity index did reach saturation, as demonstrated by the flat curve in Figure S4, suggesting that the observed sequences may function as a good representation of the bacterial communities associated in the 12 samples.

**Table 1:** Diversity indices from 12 samples

Sample	Reads	OTU		Chao1		Shan non	
		97%	95%	97%	95%	97%	95%
A	26,483	494.0	369.0	584.725	447.906	5.969	5.347
B	27,050	377.0	273.0	443.585	333.022	4.870	4.509
C	36,321	503.0	367.0	688.542	527.111	5.114	4.751
D	19,045	477.0	363.0	537.165	411.232	5.635	5.250
E	48,383	465.0	338.0	721.518	480.500	5.776	5.218
F	38,621	454.0	349.0	592.265	532.467	5.399	4.902
G	19,797	506.0	355.0	565.455	394.590	5.948	5.510
H	30,301	436.0	310.0	544.358	377.776	5.174	4.458
I	32,014	536.0	397.0	692.0	548.034	5.992	5.495
J	25,699	457.0	334.0	508.000	366.635	5.458	4.931
K	39,878	419.0	316.0	554.046	389.439	4.714	4.144
L	33,095	614.0	446.0	759.088	516.909	6.196	5.784

### Bacterial community composition and principal component analysis (PCA)

**Figure 4:**
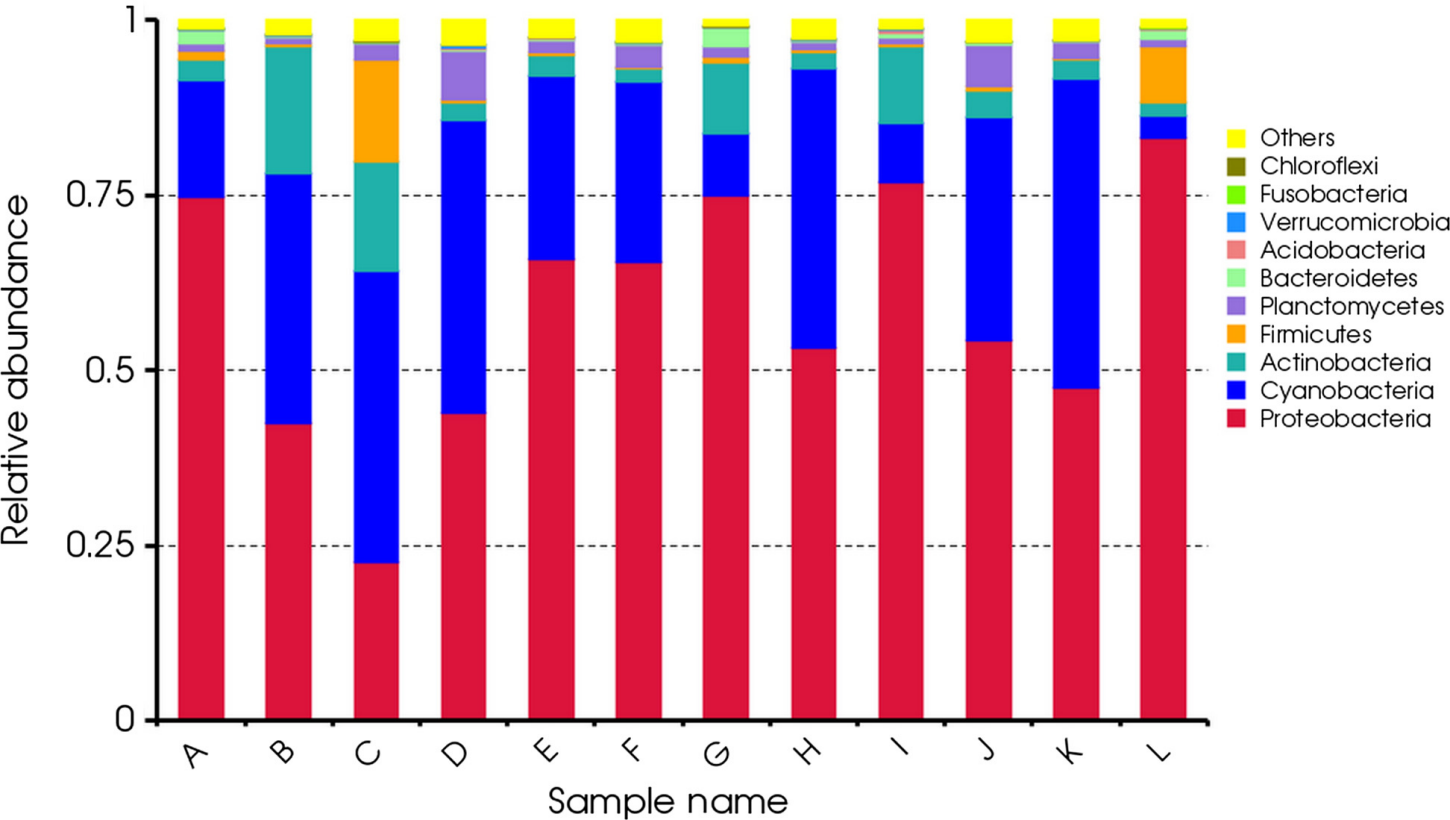
Relative abundance of bacteria in the 12 samples at the phylum level. Proteobacteria was the dominant phylum and accounted for 22.7% to 76.9% of all OTUs, followed by Cyanobacteria (3.1% to 44.2%), Actinobacteria (1.79% to 18.3%), Firmicutes (0.27% to 14.45%), and Planctomycetes (0.86% to 1.7%). The top ten phyla represented 96.45% to 99.24% of the detected bacteria.

**Figure 5:**
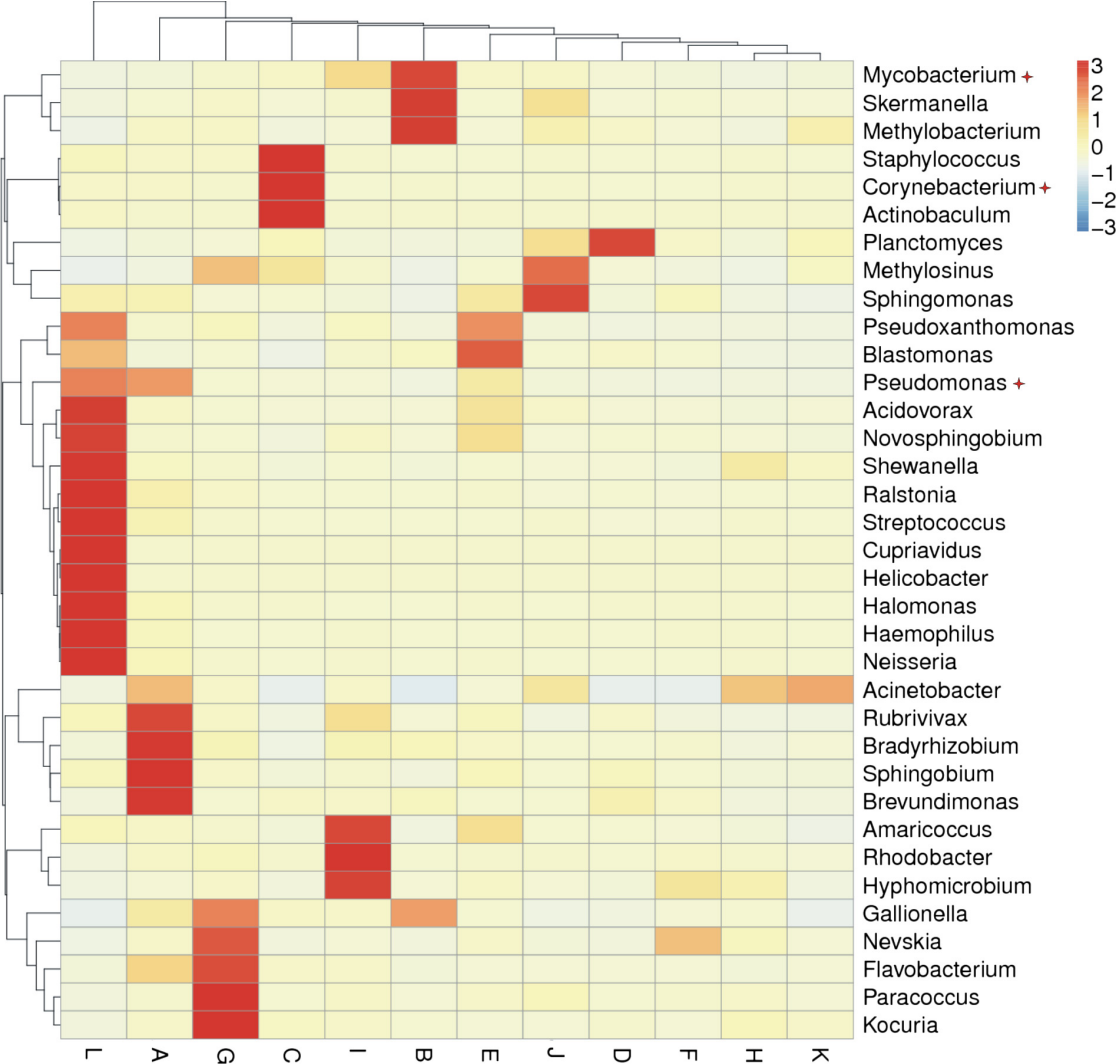
Relative abundance of each taxonomic genus. Heat map illustrates the abundance of the top 35 genera in each sample. Scale bar shows the variation of the normalized abundance. Opportunistic pathogenic bacteria were marked by 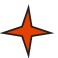.

Bacterial sequences in the 12 samples were classified into taxonomic classes using the MiSeq platform default settings. A detailed comparison of diversity and the relative abundance at the phylum level is provided in Table S1. The abundance of the top 10 phyla was shown in Figure 4. The main microbes in all samples were Proteobacteria, with the exception of sample C, which contained more Cyanobacteria (41.6%). Other dominant phyla in samples A, B, E, G, H, and K were Cyanobacteria and Actinobacteria, while Cyanobacteria and Planctomycetes dominated in samples D, F, and J. Interestingly, sample L contained the most Proteobacteria (83.2%) of the 12 samples, and sample G contained the most Bacteriodetes (2.7%). Firmicutes, a dominant phylum in the human intestine, was detected in all 12 samples, but its relative abundance was higher in samples C and L. In 12 samples, 0.51–3.42% of all detected OTUs could not be assigned to any known group, which may indicate a novel uncharacterized phylum present in Beijing drinking water. Sample D contained the highest number of unknown bacterial sequences, followed by samples J and C, while samples G and L had lower content. At the genus level, the abundance of the top 35 genera is shown in Figure 5. A detailed comparison of diversity and abundance of genus in these samples is provided in Table S2. Horizontal comparison can identify samples contained more clustered species, and the darker the color was, the more the number of the specific genus was. For example, *Mycobacterium*,* Skermanella* and *Methylobacterium* dominated in sample B, while *Kocuria*,* Paracoccus*,* Flavobacterium,* and *Nevskia* dominated in sample G. In addition, *Acidovorax,* dominated in sample L, can produce a variety of organic compounds, which can form biofilms in pipeline environments (Hong et al. 2010) and accelerate pipeline corrosion (Li et al. 2010). The microbial community of sample L was different from other samples due to different sources water. MiSeq sequencing data also revealed that potentially pathogenic bacteria occurred in all 12 samples, which are noted in Figure 5.

**Figure 6:**
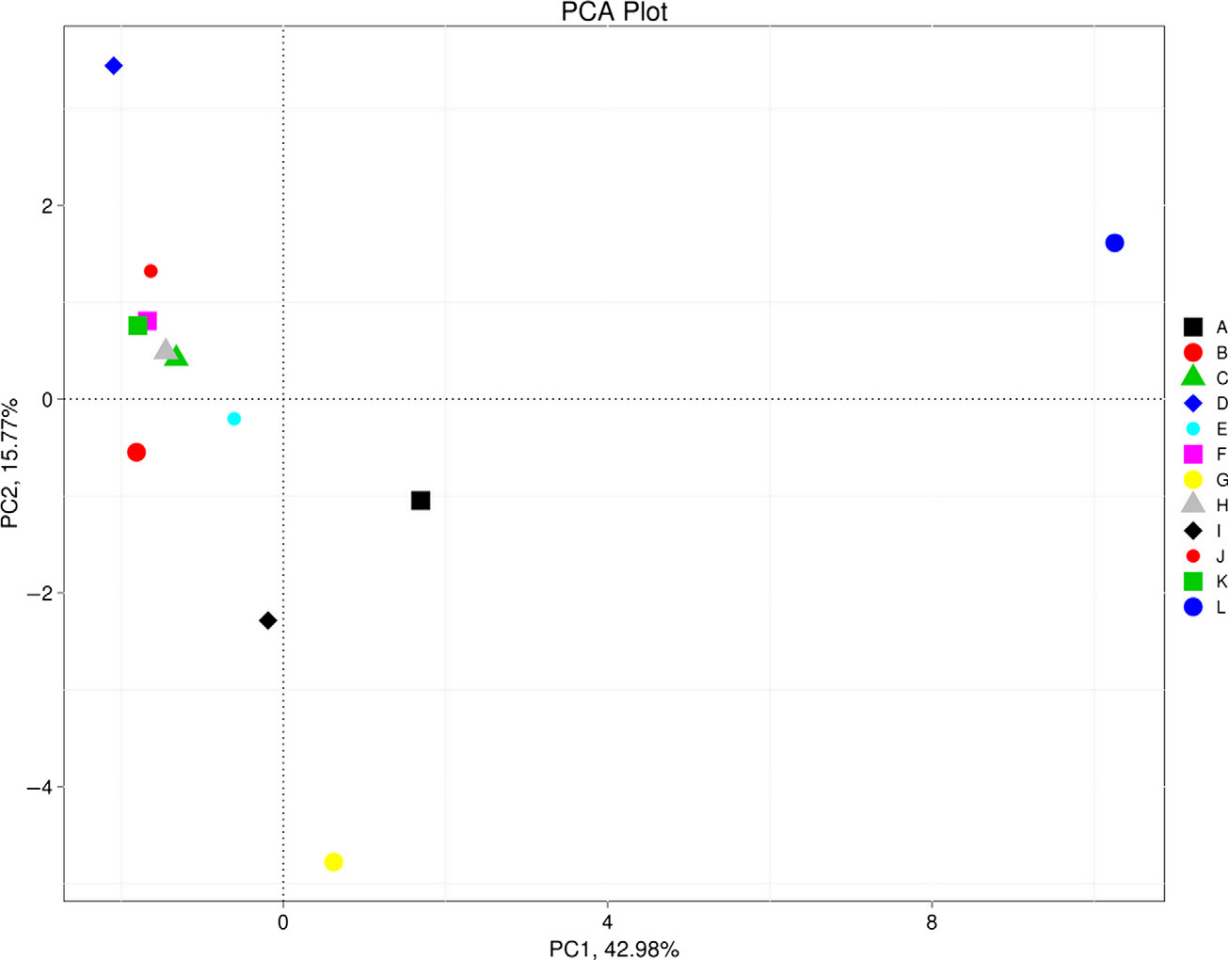
Samples sorting analysis. Scatter plot of PCA score depicting variance of fingerprints derived from different bacterial communities. Principal components (PCs) 1 and 2 explained 42.98% and 15.77% of the variance, respectively. The more similar the bacterial community, the closer the distance in the PCA score scatter plot.

Principal component analysis with weighted UniFrac distances was performed using QIIME software version 1.7.0 (Fig. 6). The PCA score plot revealed structural differences between samples. The network samples clustered closely, suggesting that samples were similar at the phylum level, although samples L was outliers, and accounted for 42.98% (PC1) and 15.77% of the variation (PC2). The reason might be that the source water of sample L was SW while others were GW. Surprisingly, sample G possessed the highest total bacterial concentration as determined by FCM, while sample D had the lowest (Fig. 2). Overall, the two PCA axes explained 58.75% of the variation between communities.

### The combination of FCM and sequencing data for quantitative characterization of potentially pathogenic bacteria

**Figure 7:**
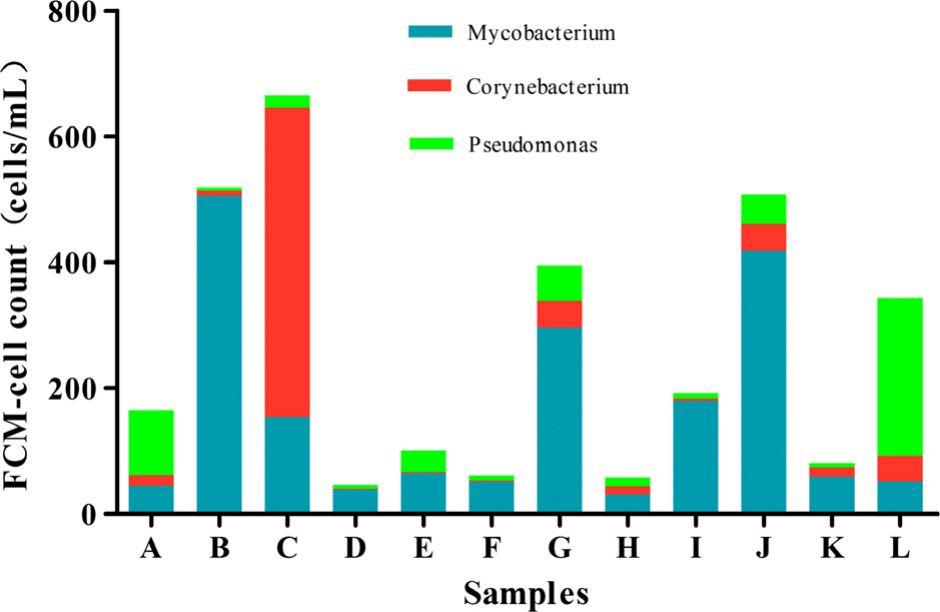
Concentrations of live opportunistic pathogenic bacteria. The value of every concentration can be acquired from the maximum minus the minimum.

Since cell concentration varied considerably between samples, comparison of pathogenic bacteria abundance at the genus level between samples was therefore limited. The absolute pathogenic bacteria concentrations were calculated, which may represent a worth noting indication of health risk. Figure 7 showed the approximately intact opportunistic pathogenic bacteria detected by calculating the total cell concentration of each sample by FCM and proportion of intact cells (Fig. 2), and the relative abundance of genera obtained by MiSeq sequencing (Table S2). For example, the FCM data indicated there were approximately 3.6 × 10^4^ total cells/mL in the sample B, 8.2% of which were viable, and the relative abundance of *Mycobacterium* was 0.17. Using these data, we could estimate that there were 500 live *Mycobacterium* per mL in the sample B. By this method (Prest et al. 2014; Shaw et al. 2015), other opportunistic pathogenic bacteria were estimated. The viable *Mycobacterium* (500 cells/mL) were highest in sample B, followed by sample J (418 cells/mL), while *Corynebacterium* (491 cells/mL) were highest in sample C. *Pseudomonas* (251 cells/mL), mainly appeared in sample L. Although sequencing does not allow to identify organisms down to the species level, most of species within these genus are pathogenic. Therefore, it may be useful to obtain approximate bacteria counts.

## Discussion

Flow cytometry (FCM) was previously proposed as an alternative to cultivation‐dependent heterotrophic plate count (HPC) to monitor microbial drinking water quality (Vital et al. 2012). In the present study, FCM was utilized to characterize indigenous aquatic bacterial communities in real water samples and detect differences between samples. HPC concentration remained stable between samples A, F and G, and was 0 CFU/mL in samples B, D and I (Fig. 2). However, these results were not consistent with the FCM data. Our findings suggest that HPC measurement sensitivity was lower than FCM when assessing small cell concentration changes such as those in samples B, C, and D. In particular, FCM intact cells resulted two to three orders of magnitude higher than HPCs due to the presence of VBNC cells, autotrophic bacteria or sublethal injury, where this difference appeared to be nonlinear across different samples. This is in agreement with observations by Foladori et al. (2015).

As seen in Figure 3, the differentiation between LNA and HNA bacteria can provide additional information. LNA bacteria are considered inactive (Phe et al. 2005) or viable and small in low‐nutrient environments (Wang et al. 2009). HNA cells are considerably larger than LNA cells (Wang et al. 2009). Several studies have shown that the HNA cluster is more dynamic and sensitive to changes than the LNA cluster, and indigenous HNA bacteria are damaged faster by chlorination (Ramseier et al. 2011). Sample G had the lowest HNA percentage (Fig. 3) and lowest number of intact cells (Fig. 2), which may indirectly reflect the degree of chlorination, consistent with previous reports (Ramseier et al. 2011). Aside from sample G, other FCM fingerprints were highly similar to the scatter plots of PCA scores, indicating the degree of similarity in bacterial community composition between samples (Fig. 6), yet FCM fingerprints only roughly reflected differences between samples.

Sequencing can further characterize in detail community structure and composition. For example, the complex biological communities of water sample treated by a four‐stage water treatment plant could be studied (Shaw et al. 2015). The differences in the composition of organic matter by different disinfectants might favor the growth of different types of bacteria, subsequently affecting the bacterial communities (Ng et al. 2015). In our study, the source water was disinfected by chlorine before distribution, resulting in a different community structure compared to the previous reports. The total number of phyla in each of the 12 samples ranged from 13 to 23, while in Beijing surface waters the number was 20 to 27 (Wei et al. 2015). Among 12 samples, the sample L contained the most phyla. The study showed that the microbial community of water originating from SW is more diverse than GW (Gomez‐Alvarez et al. 2015).Within the Proteobacteria phylum, *α*‐Proteobacteria was the most dominant class in each water sample, followed by *γ*‐, *β*‐, and *δ*‐Proteobacteria (Table S3), consistent with results from a previous study (Kwon et al. 2011) in which *α*‐Proteobacteria was more abundant than others. However, Huang et al. (2014) reported that *β*‐Proteobacteria was more abundant in drinking water, and Pinto et al. (2012) showed that *β*‐Proteobacteria (40%) was more abundant than *α*‐Proteobacteria (21%). This difference may be due to changes in water conditions, such as dissolved organic carbon (Jones et al. 2009), pH (Newton et al. 2007) and salinity gradients (Holmfeldt et al. 2009). The Firmicutes species found were mainly Bacilli and Clostridia (Table S3), which contain many spore‐producing species and are better adapted to environmental conditions that are not conducive to microbial growth, including heat, chemical solvents, oxidizers, ultraviolet radiation, and fungicides (Abecasis et al. 2013). The release of spores means that these bacteria can survive in chlorinated water. Furthermore, these species are involved in the iron cycle and pipeline corrosion (Emde et al. 1992; Kostka et al. 2002) and thus capable of utilizing a variety of substrates (Newton et al. 2011).Potentially pathogenic bacteria in drinking water distribution systems may be a cause for alarm. *Mycobacterium* are very complex and generally resistant to disinfectants (Liu et al. 2012b). Biofilm formation and amoeba‐associated lifestyle have been recognized as important factors that contribute to the survival, colonization and persistence of *Mycobacterium* in water distribution systems (Vaerewijck et al. 2005). Biofilms microorganisms were known to be more resistant to drinking water disinfection than free‐living microorganisms (Lewis 2001). Atypical *Mycobact*e*ria* can cause a range of diseases involving the skeleton, lymph nodes, skin, and soft tissues. Detections of atypical *Mycobacteria* in drinking‐water and the identified routes of transmission suggested that drinking‐water supplies were a plausible source of infection (Organization, 2004). Many of *Corynebacterium* were associated with secondary infections in patients where defense mechanisms have been weakened by primary infections caused by more virulent pathogens (Organization, 2004). *Corynebacterium* and *Mycobacterium*, as waterborne pathogens, were identified in various postmonochloramine water (Shaw et al. 2015).*Pseudomonas* was identified as potential pathogens in a drinking water treatment membrane filtration system (Kwon et al. 2011). After pipeline distribution, it still present in the tap water (Huang et al. 2014). For example, *P. aeruginosa* grow on drinking water biofilms (Moritz et al. 2010), and only certain specific hosts were at risk, including patients with profound neutropenia, cystic fibrosis, severe bums (Hardalo and Edberg 1997). Intake of drinking‐water is not an important source of infection by *P. aeruginosa*. But susceptible tissue, notably wounds and mucous membranes exposed in water containing *P. aeruginosa* can cause a range of diseases (Organization, 2004). In short, pathogenic bacteria were present at relatively high concentrations in samples B, C, J, G, and L, indicating that more attention should be paid to these sampling sites in the future. However, the identity of pathogenic species sequences was relatively low, although more sequences were assigned into several genera containing pathogenic species, suggesting that comparison at the genus level may overestimate the pathogenic populations (Ye and Zhang 2011).

The classical microbiological methodology relies on plate counts, which makes assessing the results difficult, and may result in data that are unrepresentative and biased and do not reflect the water pathogen content or identity (Szewzyk et al. 2000). In this study, the methods of flow cytometry and 16S rRNA gene sequencing were combined for the detection and quantification of opportunistic pathogenic bacteria groups, which were not only qualitative but also quantitative. By this research, we can envision that flow cytometry and sequencing may replace existing culture‐based methods which were time consuming and can only target specific species and become standard tools in the future for drinking water quality monitoring.

## Conclusions

Studies on microbial community characteristics in drinking water distribution systems using FCM and 16S rRNA gene sequencing showed the following: (1) FCM and MiSeq sequencing enable the detection of water microbial community features that are not detected by classical methods such as HPC; (2) 16S rRNA gene sequencing provided insight into the bacterial community composition and revealed differences in bacterial communities between 12 water samples taken from different locations; (3) the combination of data obtained from these two methods provides quantitative information on potential pathogenic bacteria that cannot be obtained by any method alone.

## Conflict of Interest

None declared.

## Supporting information


**Figure S1.** FCM results are represented as dot‐plots of total cell counts, and low nucleic acid content (LNA) bacteria and high nucleic content acid (HNA) bacteria are indicated using fixed electronic gates.

**Figure S2.** Schematic of 100‐L hollow‐fiber ultrafiltration experimental setup.

**Figure S3.** Rarefaction curves for a dissimilarity of 3% from 12 samples.

**Figure S4.** Shannon diversity index curves.

**Table S1.** Abundance at the phylum level.

**Table S2.** Abundance at the genus level.

**Table S3.** Abundance at the class level.
