# Supplementary figures and images for: Bacterial characterization of Beijing drinking water by flow cytometry and MiSeq sequencing of the 16S rRNA gene
Source: Ecol Evol. 2016 Jan 18;6(4):923–34. doi: 10.1002/ece3.1955 (PMC4761785; doi:10.1002/ece3.1955)

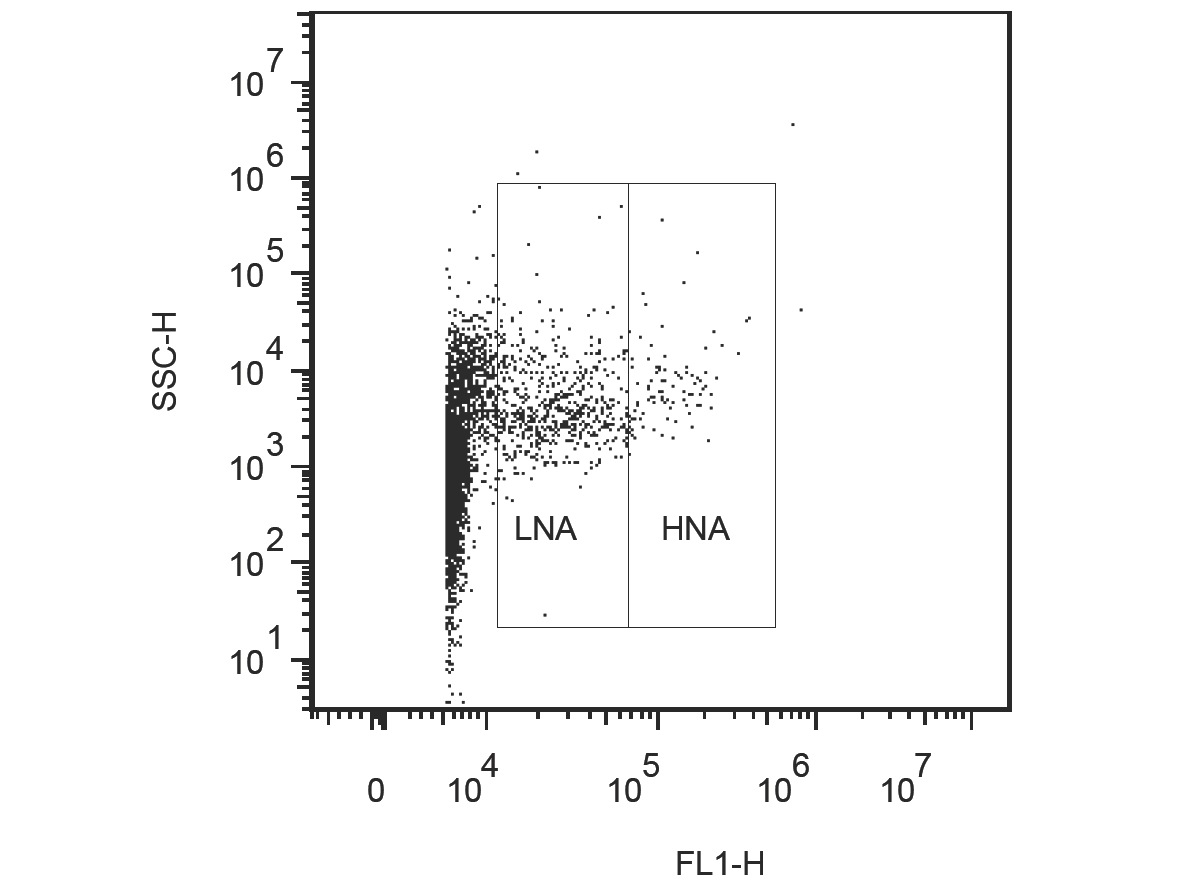

Supplement: Supplementary file 1 — Figure S1. FCM results are represented as dot‐plots of total cell counts, and low nucleic acid content (LNA) bacteria and high nucleic content acid (HNA) bacteria are indicated using fixed electronic gates. [file ECE3-6-0923-s001.tif]

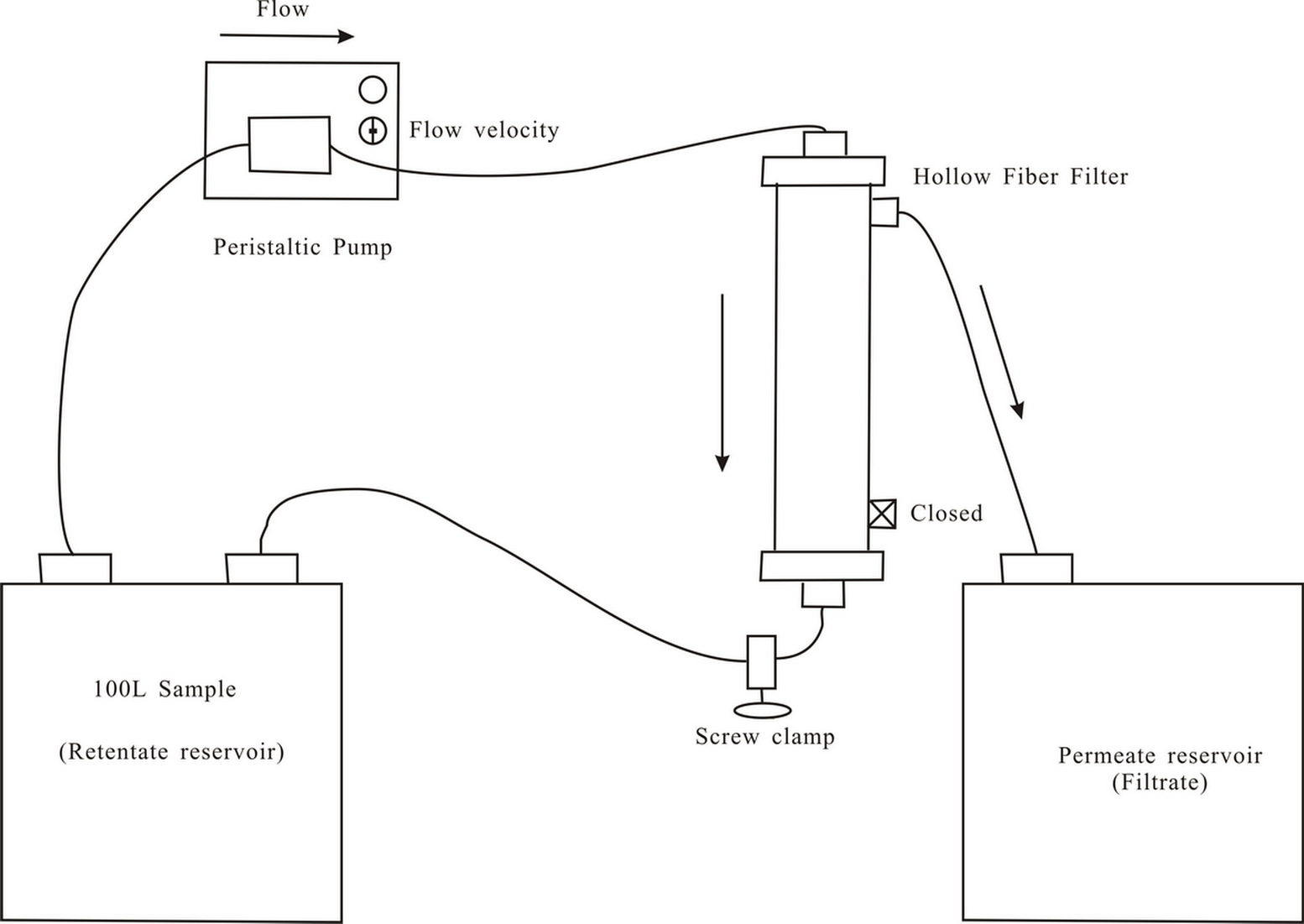

Supplement: Supplementary file 2 — Figure S2. Schematic of 100‐L hollow‐fiber ultrafiltration experimental setup. [file ECE3-6-0923-s002.tif]

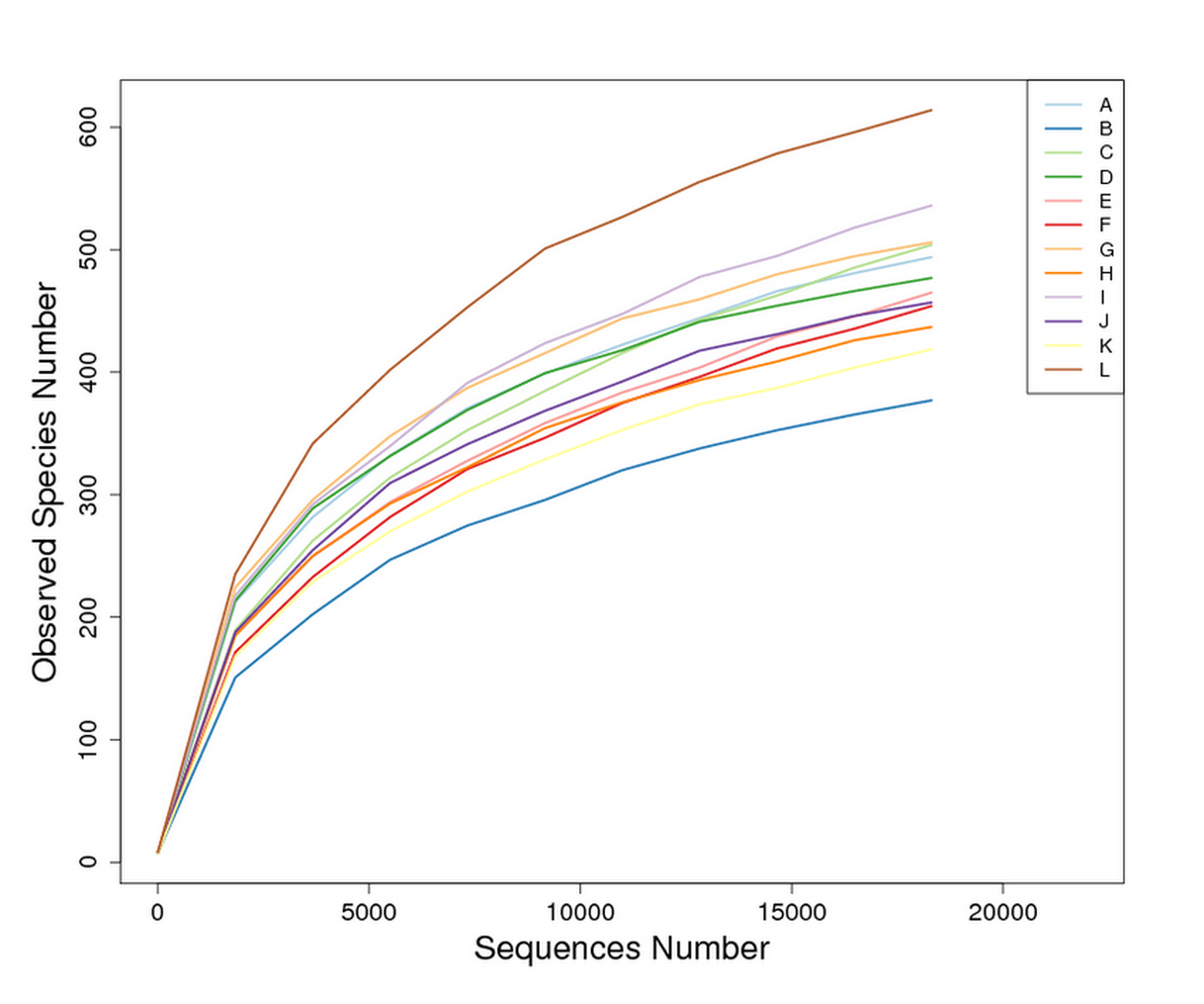

Supplement: Supplementary file 3 — Figure S3. Rarefaction curves for a dissimilarity of 3% from 12 samples. [file ECE3-6-0923-s003.tif]

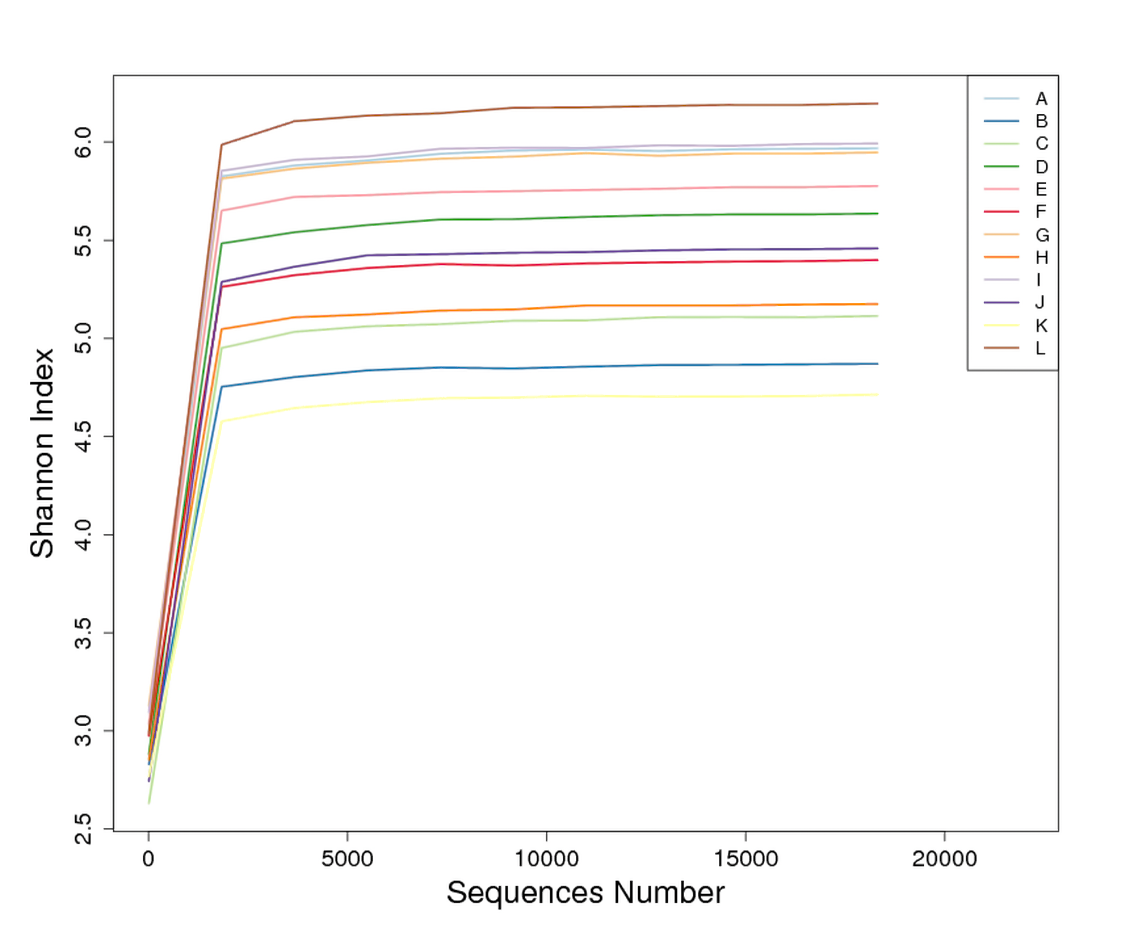

Supplement: Supplementary file 4 — Figure S4. Shannon diversity index curves. [file ECE3-6-0923-s004.tif]
